# MicroRNA and Putative Target Discoveries in Chrysanthemum Polyploidy Breeding

**DOI:** 10.1155/2017/6790478

**Published:** 2017-12-13

**Authors:** Fengjiao Zhang, Jingya Zhao, Sujuan Xu, Weimin Fang, Fadi Chen, Nianjun Teng

**Affiliations:** College of Horticulture, Nanjing Agricultural University, Key Laboratory of Landscaping Agriculture, Ministry of Agriculture, Nanjing 210095, China

## Abstract

MicroRNAs (miRNAs), around 22 nucleotides (nt) in length, are a class of endogenous and noncoding RNA molecule that play an essential role in plant development, either by suppressing the transcription of target genes at a transcriptional level or inhibiting translation at a posttranscriptional level. To understand the roles of miRNAs and their target genes in chrysanthemum polyploidy breeding, three sRNA libraries of normal and abnormal embryos after hybridization were performed by RNA-Seq. As a result, a total of 170 miRNAs were identified and there are 41 special miRNAs in cross of paternal chromosome doubling, such as miR169b, miR440, and miR528-5p. miR164c and miR159a were highly expressed in a normal embryo at 18 days after pollination, suggesting the regulatory role at the late stage of embryonic development. miR172c was only detected in the normal embryo at 18 days after pollination, which means that miR172c mainly mediates gene expression in postembryonic development and these genes may promote embryo maturation. Other miRNAs, including miR414, miR2661, and miR5021, may regulate the genes participated in pathways of auxin response and energy metabolism; then they regulate the complex embryonic development together.

## 1. Introduction

In plants, microRNAs (miRNAs) are a major class of small noncoding RNAs (sRNAs) with 20–22 nt. sRNAs have been identified to control the developmental processes in plants by regulating gene expression [1]. They have the potential to regulate a gene by two ways: (1) posttranscriptional gene silencing (PTGS) by binding to 3′ untranslated region (UTR) of messenger RNAs (mRNAs) and repressing the translation of target mRNAs; (2) transcriptional gene silencing (TGS) by epigenetic modifications [2, 3]. PTGS is the main strategy used by miRNAs to regulate gene expression in plant development. Plant mature miRNAs are generated from miRNA precursors that are processed by a ribonuclease DICER-LIKE1, which negatively regulates specific target mRNAs [2].

A number of studies have investigated the key regulatory role of miRNAs in a wide range of growth and development processes in plants, including the regulation of embryonic development [4, 5]. Plant embryo development includes two stages, embryo morphogenesis and seed maturation. In the model plant *Arabidopsis thaliana*, embryonic pattern formation has been mainly concerned and studied [5]. Based on the embryo shape, the cell division goes through several stages from preglobular to mature embryo, tightly regulated by multiple genes [6]. As a class of small regulatory RNA, the function of miRNAs during plant embryo development has been considerably reported recently [7]. 28,645 mature miRNAs have been discovered and deposited in the public miRNA database miRBase (Release 21, http://www.mirbase.org/) and hundreds of miRNA target in plant embryo. A large number of *Arabidopsis* mutants of miRNA biogenesis genes have revealed the crucial roles of miRNA during seed morphogenesis and maturation. Willmann et al. reported the earlier timing of embryo maturation in *Arabidopsis* mutant for strong alleles of *DCL1* (*DICER-LIKE1*) that are required for miRNA biogenesis and demonstrated the negative regulatory role of specific miRNAs during early embryogenesis and later in embryonic development and that miRNAs are key regulators during seed maturation program [8].

Embryonic miRNAs mediate plant embryo development by regulating transcription factors located in special spatial organization and other key developmental regulators [5]. In plants, miR165/166 is one of the best characterized miRNA families, which regulates five class III homeodomain leucine zipper genes (*HD-ZIP IIIs*) (*PHB*, *PHV*, *REV*, *CNA*, and *ATHB8*) [9, 10]. The HD-Zip III gene family regulates apical embryo patterning and organ polarity as well as controls shoot and root apical meristem (SAM and RAM) formation in embryogenesis [11–13]. The family of miR160/miR167 regulates the target mRNAs of auxin response factors (ARFs) associated with auxin homeostasis [14, 15]. Auxin response is an important signaling pathway during embryonic pattern formation, embryo development, and seed maturation [16].

Chrysanthemum (*Chrysanthemum morifolium*) is an economically important flower around the world, with the increase of chrysanthemum consumption, breeders are driven to improve the cultivars' traits, such as color, size, shape, and tolerance [17]. Artificial distant hybridization is one of the most effective methods to improve and create new cultivars. However, embryo abortion commonly happens during hybridization [18, 19]. Although previous studies analyzed many different reasons leading to plant embryo abortion, including maternal genotypes [20], parent ploidy [21, 22], and gene regulation [6, 23], the molecular mechanisms regulating embryo development are poorly understood. Zhang et al. revealed the gene, protein, and miRNA change in the stage of chrysanthemum embryo development [19, 24] and provided numerous information of embryo abortion in chrysanthemum hybridization breeding. Evidence supporting a major role for chromosome doubling in overcoming chrysanthemum embryo abortion has been obtained from some studies [22, 25], but the functions of miRNA remain largely uncharacterized.

Next generation sequencing (NGS) approaches have been employed to identify individual miRNAs in various samples, and bioinformatics analyses have offered the technical support to predict the miRNA targets [26]. In the present study, we sequenced three small RNA libraries from chrysanthemum embryo in cross *C. morifolium* “Yuhualuoying” × tetraploid *C. nankingense* and identified the key miRNAs and targets that may facilitate embryo development in cross of paternal chromosome doubling.

## 2. Materials and Methods

### 2.1. Plant Materials and Artificial Hybridization

Artificial hybridization was performed in cross *C. morifolium* “Yuhualuoying” (♀, 2n = 6X = 54) × tetraploid *C. nankingense* (♂, 2n = 4X = 36). Here, the male is an autopolyploid generated by colchicine doubling of the diploid *C. nankingense* (2n = 2X = 18) [19]. After pollination, three samples were collected, corresponding to the normal embryo at 12 days after pollination (DAP) (NE12), normal embryo at 18 DAP (NE18), and abnormal embryo at 18 DAP (AE18). For each sample, we collected 0.2 g independent biological replicates for verification and mixed the rest of triplicate samples (~0.5 g) for RNA-Seq. All of the samples were immediately frozen in liquid nitrogen and stored at −80°C.

### 2.2. RNA Extraction, Small RNA Library Preparation, and Illumina Sequencing

Total RNA was extracted with TRIzol reagent (Takara Bio Inc., Otsu, Japan) according to the manufacturer's protocol. Small RNAs with 18–30 nt fragments were enriched by 15% denaturing polyacrylamide gel electrophoresis. After purification, they were ligated to 5′ and 3′ adaptors and reversed transcribed into cDNA by reverse transcription-PCR (RT-PCR). Three small RNA libraries were constructed and sequenced using the Illumina HiSeq™ 2000 by the Beijing Genomics Institute (BGI) (Shenzhen, Guangdong Province, China).

### 2.3. Conserved and Novel miRNA Prediction

Firstly, the data cleaning analysis was performed by getting rid of low-quality reads, reads with 5′ primer contaminants and poly A, reads without 3′ primer and the insert tag, and reads shorter than 18 nt. The small RNA tags with miRNA, rRNA, snRNA, snoRNA, and tRNA were annotated by aligning to GenBank (http://www.ncbi.nlm.nih.gov/genbank/) and Rfam database (http://rfam.xfam.org/) using all clean reads of 18~30 nt. The number and proportion of each type of sRNAs were calculated in three libraries. Then, to identify the miRNAs in chrysanthemum embryo, miRBase 19.0 (http://www.mirbase.org/) was used to search the conserved miRNAs by BlantN. Only 90% matched sequences were considered to be conserved miRNAs. To allow the unambiguous mapping of small RNAs to annotations, the priority rule was followed: rRNA (in which GenBank > Rfam) > known miRNA > repeat > exon > intron. Finally, the novel miRNAs were predicted using the Mireap software (https://sourceforge.net/projects/mireap/); here, the chrysanthemum embryo transcriptome library obtained from the same sample with the present study (the NCBI accession number PRJNA315793) was used as the reference database. In the chrysanthemum embryo, a total of 99,119 unigenes were assembled with a mean length of 550–580 nt, which were used for prediction in the present study.

### 2.4. Target Prediction and Functional Annotation for miRNAs

The potential target genes of the known miRNAs were predicted by the web tool psRNATarget (http://plantgrn.noble.org/psRNATarget/) with parameters suggested by Allen et al. [27]. The target genes were identified in chrysanthemum embryo transcriptome dataset [22], and the function of these potential target genes was annotated using the two protein databases, Gene Ontology (GO) (http://geneontology.org/) and Kyoto Encyclopedia of Genes and Genomes (KEGG) (http://www.genome.jp/kegg/).

### 2.5. Differential Expression of Known miRNA and Their Targets

To find out the differentially expressed miRNAs in three samples, we normalized the expression of miRNA in three samples and obtained the expression of transcript per million (TPM). Normalized expression = actual miRNA count/total count of clean reads^∗^1000000. If the miRNA count was zero, it was revised to 0.01 for analysis of differential expression. The fold-change of log_2_ (sample 1/sample 2) and *p* value from the normalized counts were calculated to determine significant expression changes. Finally, those miRNAs with fold-change > 1 and *p* value <0.05 were considered to be differentially expressed in the two samples. Heatmap is a visual tool reflecting the expression of differential miRNAs. In the present study, the OmicShare tool, a free online platform for data analysis (http://www.omicshare.com/tools), was plotted. Based on the transcriptome library, we searched the expression pattern of these target genes regulated by the differentially expressed miRNAs.

### 2.6. Real-Time Quantitative PCR (qRT-PCR) Validation of miRNAs and Target Genes

We randomly selected 12 miRNAs and 9 target genes to validate the reliability of sRNA sequencing by qRT-PCR. RNA samples used for sequencing were reverse transcribed using PrimeScript miRNA qPCR starter kit ver 2.0 (Takara Bio, Dalian, China). qRT-PCR was performed using the SYBR Premix EX Taq Kit (Takara, Dalian, China), and the PCR amplification was done as described by Zhang et al. [24]. Three biological replicates were performed for each sample, and relative expression levels were calculated by the 2^−△△CT^ method. The chrysanthemum gene *EF1α* (*elongation factor 1a*) (GenBank accession number KF305681) was used as a reference, which is stably expressed in chrysanthemum [19]. Special primers were designed using PRIMER3 RELEASE 2.3.4. All of the primers were shown in Table S1.

## 3. Results

### 3.1. Sequencing Analysis of sRNA

To explore the regulation of miRNAs in chrysanthemum embryo development when paternal chromosomes were doubled, three cDNA libraries of small RNAs were constructed, which were named NE12, NE18, and AE18. All clean reads were obtained by filtering the low-quality sequences, adapter sequences, and poly-A sequences shorter than 18 nt, which altogether resulted in more than 99.79% of raw reads. When these small RNA tags were mapped to genome, about 20% of reads in each sample were matched. The unique sRNAs matched to genome in the three libraries were 3,864,037, 3,780,667, and 3,734,164, accounting for 9.62%, 10.04%, and 10.29% of all unique sRNAs (Table 1).

**Figure 1:**
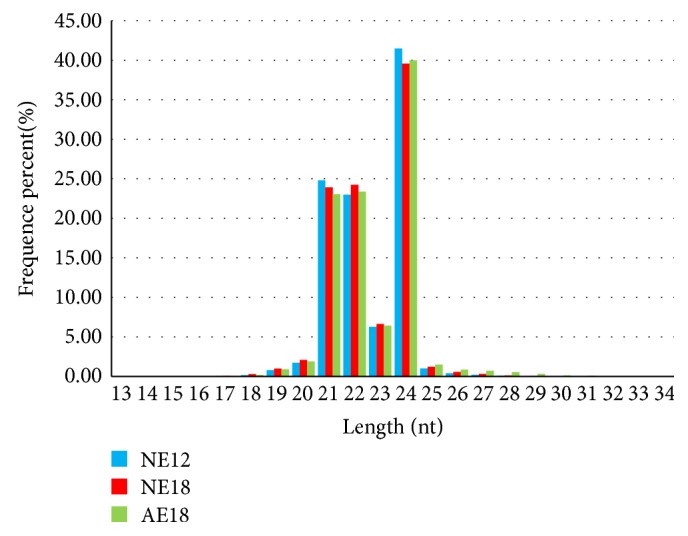
Length distribution of sRNAs in chrysanthemum embryo. *x*-axis is the length of sRNA distribution, and *y*-axis is the proportion of the sRNAs of different lengths. NE12, NE18, and AE18 mean the normal embryo at 12 DAP (days after pollination), normal embryo at 18 DAP, and abnormal embryo at 18 DAP, respectively.

When these unique sRNAs were aligned to the GenBank and miRBase, many types of sRNAs were identified, including miRNA, rRNA, snRNA, snoRNA, and tRNA, but the vast majority of sequences were unannotated. The length of different types of sRNAs was discrepant; since in chrysanthemum embryo, the most abundant classes of sRNA showed the length of 24 nt (dominant siRNA), then 21 nt (mainly miRNAs), and 22 nt (Figure 1). In the present study, the unique miRNAs in each library were taken into consideration for subsequent analysis, and the proportion of miRNAs was 0.26%, 0.24%, and 0.26% in libraries of NE12, NE18, and AE18 (Table 1).

### 3.2. miRNAs and Target Genes Identified in Three Libraries

**Figure 2:**
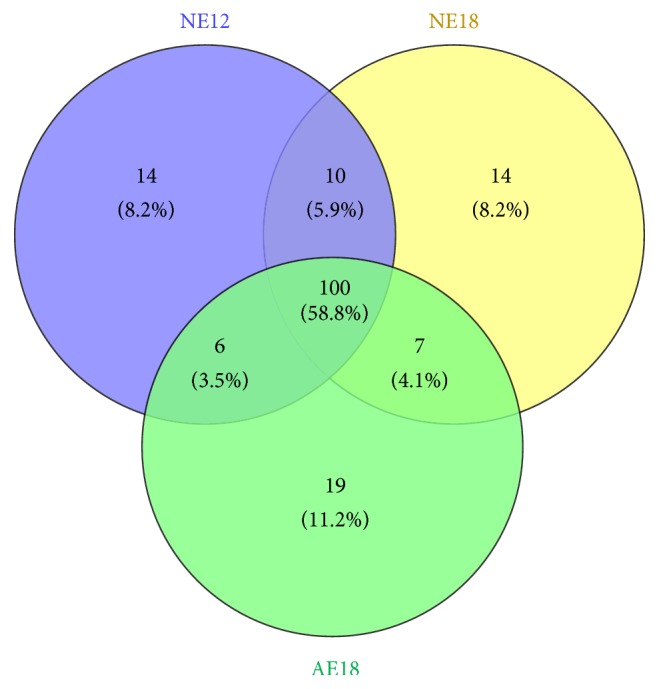
Venn diagram presented the quantity distribution of conserved miRNAs in chrysanthemum embryos. There were 130, 131, and 132 miRNAs expressed in NE12, NE18, and AE18, respectively. In the middle, 100 means that the number of miRNAs was detected in three samples. NE12, NE18, and AE18 mean the normal embryo at 12 DAP, normal embryo at 18 DAP, and abnormal embryo at 18 DAP, respectively.

In all samples, a total of 170 conversed miRNAs were identified in miRBase, and 130, 131, and 132 miRNAs were expressed in NE12, NE18, and AE18, respectively. 100 miRNAs (accounting for 58.8%) were detected in three samples; however, some were in two different samples, such as 10 miRNAs in NE12 and NE18, 7 miRNAs in NE18 and AE18, and 6 miRNAs in NE12 and AE18. Venn diagram (Figure 2) presented the quantity distribution of conserved miRNAs in chrysanthemum embryo.

Expression level is an important feature to explain the regulation function of miRNAs, and they commonly varied greatly in different samples. Here, the expression of 170 miRNAs with sequences was normalized and analyzed. The most abundant miRNAs in three samples were miR156a, miR157a, and miR166a, with the expression more than a thousand (Table S2). However, some miRNAs highly expressed in a particular sample, such as the expression of miR398b-5p, are 3524 in AE18 but neither in NE12 nor NE18, miR5721 only expressed in NE18, and miR5662 only in NE12.

To study the biological function of miRNA in chrysanthemum embryo development, the sequences of target mRNA were paid attention. A total of 770 target genes were identified and regulated by 88 miRNAs (51.76% of all miRNAs), and miRNA414 had the most targets (347 unigenes), followed were miR5293 (61 targets) and miR5021 (44 targets) (Table S3). 34 target genes were regulated by two miRNAs, such as CL1999. Contig2 was targeted by miR165a and miR166a; unigene10412 was the target of miR156a and miR157a (Table S3).

### 3.3. Functional Annotation of Target Genes

**Figure 3:**
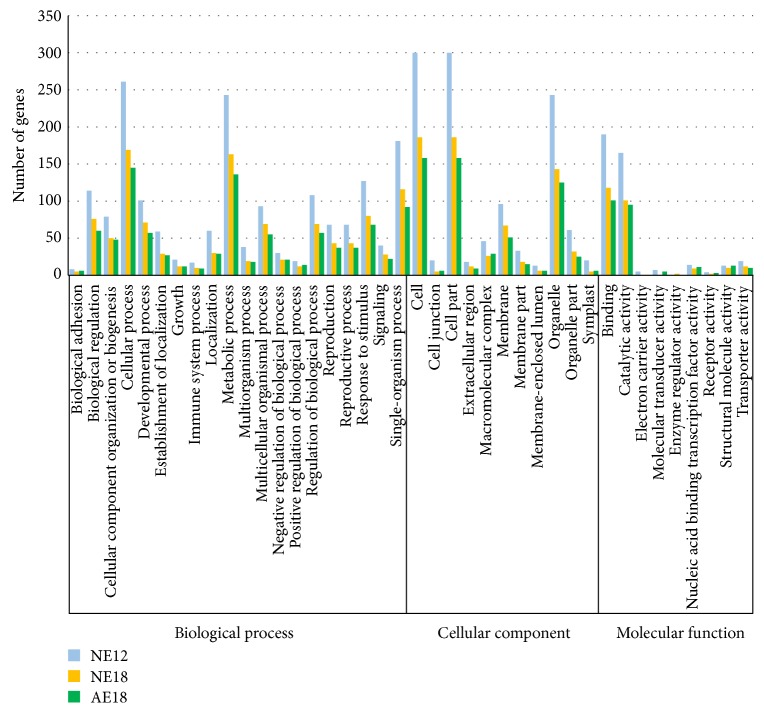
GO classification of target genes in three samples. *x*-axis means three categories of GO: biological process, cellular component, and molecular function. *y*-axis is the number of genes in three samples associated with each subcategory. NE12, NE18, and AE18 mean the normal embryo at 12 DAP, normal embryo at 18 DAP, and abnormal embryo at 18 DAP, respectively.

Gene Ontology (GO) is a standardized classification system of gene used to describe the characteristics of genes and gene products in organisms. The result showed that 517 target genes were classified into three gene ontology categories: biological processes, cellular components, and molecular functions (Figure 3). Of the 40 functional categories, the number of genes in each sample is different, but in most of categories, NE12 had the largest number but least in AE18. In the third ontology of molecular function, some target genes were expressed in a specific sample, such as the genes related to “electron carrier activity” only expressed in NE12, two genes in the category of “enzyme regulator activity” expressed in NE18, but there was no gene regulated “molecular transducer activity” in NE18 (Figure 3).

To understand the biological function of target genes, KEGG analysis, as with GO, was also used to analyze candidate targets. In NE12, a total of 337 target genes had the biological function on 207 KEGG pathways, but there were less target genes and pathways in NE18 and AE18. 211 target genes with 118 pathway annotation in NE18 and 179 annotated target genes with 113 pathways in AE18 (Table S4). Some target genes were annotated in dozens of pathways that are only in library of NE12, including some energy metabolism pathways, such as ko00280 (valine, leucine, and isoleucine degradation), ko00310 (lysine degradation), and ko00410 (beta-alanine metabolism).

### 3.4. Differentially Expressed miRNAs in Chrysanthemum Embryo

**Figure 4:**
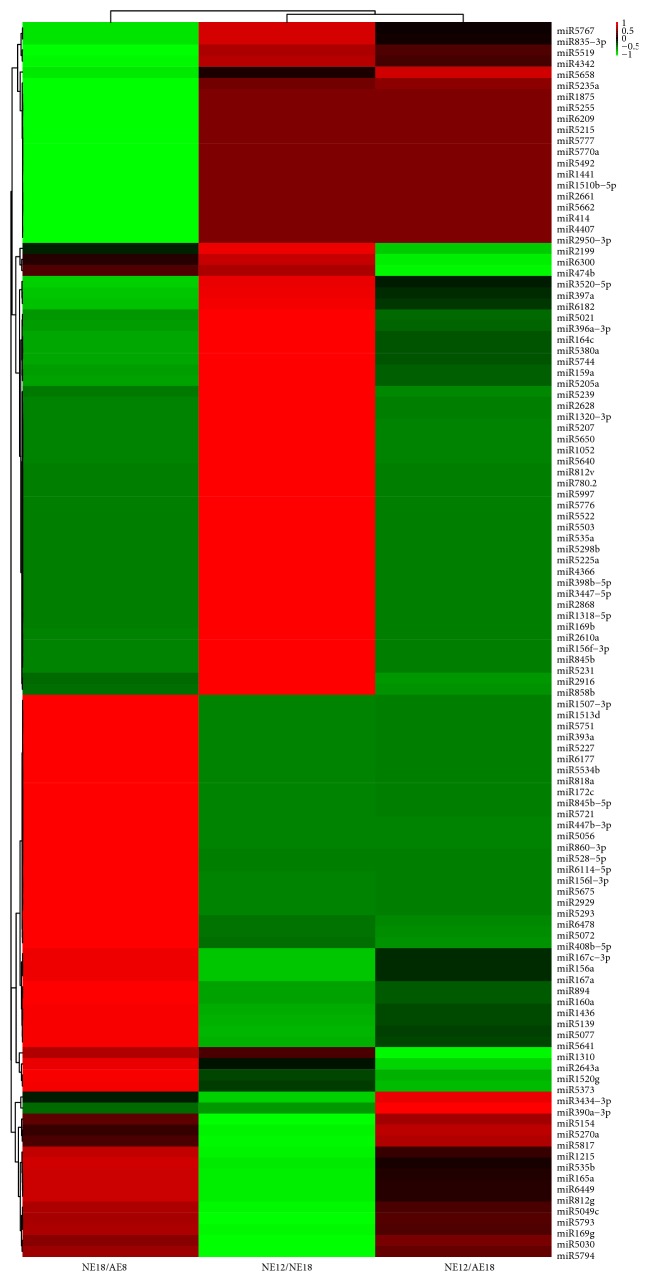
Heatmap showed the expression pattern of differentially expressed miRNAs in chrysanthemum embryos. The bar represents the ratio of expression levels of miRNAs between two samples. Three ratios of NE18/AE18, NE12/NE18, and NE12/AE18 indicated the expression pattern of each miRNA in three samples. Red rectangle means upregulation, and green means downregulation. All information for each miRNA list can be found in Table S2. NE12, NE18, and AE18 mean the normal embryo at 12 DAP, normal embryo at 18 DAP, and abnormal embryo at 18 DAP, respectively.

The aim of this study is to analyze the different miRNAs possibly involved in chrysanthemum embryo development, so we identified 112 differentially expressed miRNAs with at least 1.5-fold after standardized expression. The heatmap showed the expression pattern of these miRNAs (Figure 4). They were assembled in three groups depending on the expression trend. In group NE18/AE18, the number of upregulated miRNAs was almost the same as downregulated, and similar quantity distribution occurred in NE12/NE18. However, the difference is in NE12/AE18, in which only 1/3 of the miRNAs were upregulated in NE12 and means more negative genes in AE18.

### 3.5. Characteristics of Target Genes in Chrysanthemum Embryo Development

**Figure 5:**
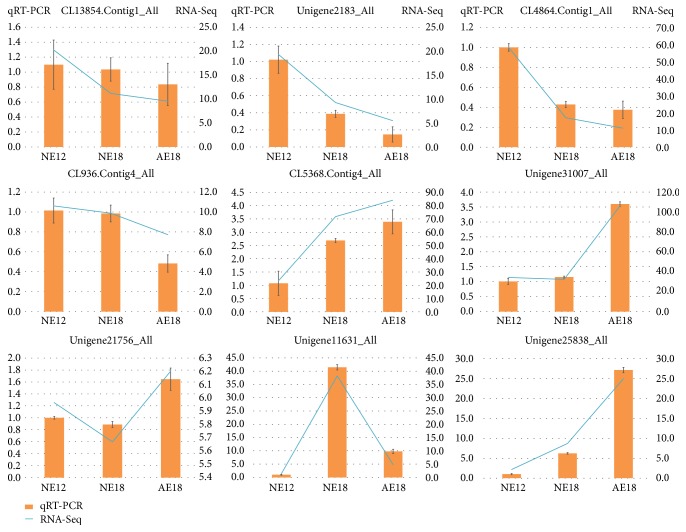
Validation of target genes in chrysanthemum embryos by qRT-PCR. The orange histogram represented the qRT-PCR validation, and the value of relative expression was shown on the left of *y*-axis. The blue slope lines represented the FPKM of each unigene obtained using RNA-Seq [19], and the value of FPKM was shown on the right of *y*-axis. NE12, NE18, and AE18 mean the normal embryo at 12 DAP, normal embryo at 18 DAP, and abnormal embryo at 18 DAP, respectively.

miRNAs regulate the plant development by mediating the expression of target genes. Apart from those redundancy regulated by several miRNAs, a total of 770 target genes were identified and annotated by chrysanthemum transcriptome dataset. The expression level and annotation were presented in Table S5. Most of them were regulated by one miRNA, and some were negatively regulated by miRNAs, such as the transcription factor MYB11 (Unigene2183) highly expressed in NE12, but the targeted miR858b was the lowest expression level in it. Another transcription factor WRKY48 (Unigene25838) was expressed to be the highest in AE18 and lowest in NE12, showing the negative regulation by miR414 (Figure 5, Tables S2 and S5). Several targets were regulated by two miRNAs, and there was no difference in the level of expression among samples. For example, unigene21756 was the target of miR5215 and miR5373, and its FPKM is near the three samples, without negative regulation by these two miRNAs.

In order to study the target genes that regulate the embryo development, some differentially expressed target genes (the fold of FPKM > 1.5) were selected according to their Nr/Nt annotation, which may be involve in chrysanthemum embryo development (Table 2). They contained some transcription factor, genes related to energy metabolism and protein synthesis, and some uncharacterized protein. The expression of transcription factor was various; WRKY and NAC were highest in AE18, but MYB was lowest. Some of the genes associated with auxin and ATP synthesis were downregulated in AE18 (Table 2 and Figure 5).

### 3.6. Validation of qRT-PCR

**Figure 6:**
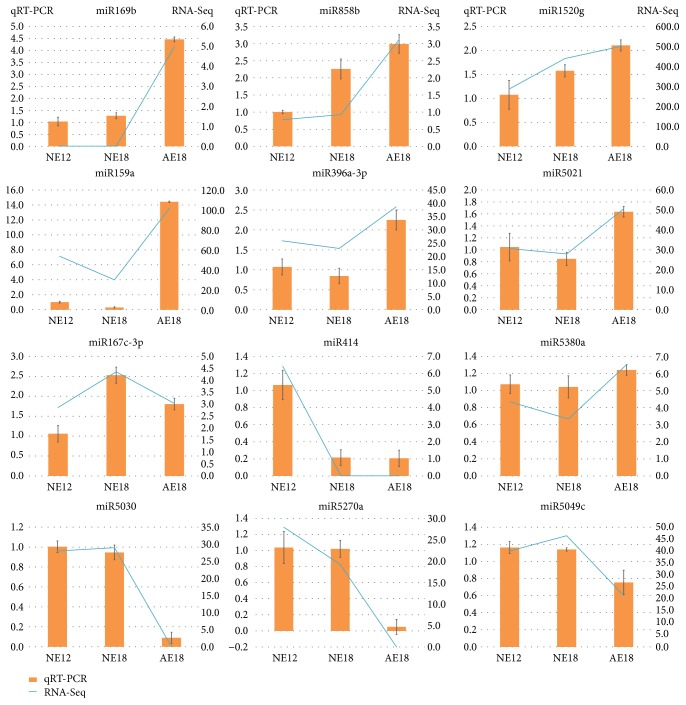
Validation of miRNAs in chrysanthemum embryos by qRT-PCR. The orange histogram represented the qRT-PCR validation, and the left of *y*-axis was the value of relative expression in three samples. The blue slope lines represented the expression level (on the right of *y*-axis) detected by RNA-Seq. NE12, NE18, and AE18 mean the normal embryo at 12 DAP, normal embryo at 18 DAP, and abnormal embryo at 18 DAP, respectively.

qRT-PCR is an efficient and accurate way to examine the result of RNA-Seq. In the present study, a total of 12 miRNAs were double tested by qRT-PCR, suggesting the high compatibility of the expression pattern between the two methods (Figure 6). Seven of them had the highest expression in AE18, such as miR169b, miR159a, and miR858b. miR167c-3p was verified with the highest expression in NE18 by two methods. miR414 that regulated the most target genes with various expression levels was expressed dominantly in NE12 (Figure 6).

## 4. Discussion

### 4.1. Identified miRNAs in Chrysanthemum Embryo

miRNAs can regulate the plant embryonic development, which important and diverse roles have been studied in various species; however, the specific function of individual miRNA is still uncharacterized for stage-specific embryo [5]. Next-generation sequencing makes it easier to identify individual miRNA families from different organs or treated plants. In *Pinus taeda* [15] and *Brassica napus* [28], miRNAs were identified from zygotic embryos at late developmental stages. More and more researchers have provided abundant evidences that miRNAs are required for the majority of embryonic cell differentiation and development in *Arabidopsis* [29, 30]. In chrysanthemum cross breeding, embryonic development is a crucial stage for seed formation, but embryo abortion is prevalent in chrysanthemum distant hybridization [31, 32]. Previous studies explored this barrier at the level of cell structure, gene expression, and miRNA regulation [19, 24], in which 227 miRNAs were identified in hybrid embryos from cross *C. morifolium* and diploid *C. nankingense*. Because of the importance of chromosome doubling in cross breeding, in the present study, we performed the hybridization using *C. morifolium* and tetraploid *C. nankingense*; 179 miRNAs were identified in three embryo samples (Table S2). Compared with two crosses, less miRNAs were identified after paternal chromosome doubling; as a result, 135 miRNAs were identified simultaneously in two crosses, 44 new miRNAs in the present cross, such as miR169b, miR440, and miR528-5p, but 92 miRNAs expressed in the previous cross were not detected here, such as miR172a, miR172b, and miR391 [24]. These similarities and differences suggest that most of the identified miRNAs in two crosses are the main factor regulating embryonic development in chrysanthemum and those expressed in specific cross may regulate the development depending on whether the male chromosome doubled. To some extent, it implicated that chromosome doubling has an effect on embryo development in distant hybridization by regulation of miRNAs.

### 4.2. The Role of miRNA Chrysanthemum Embryo

In plant embryo, miRNAs can mediate their downstream targets and regulate the expression of some transcription factors or other key developmental regulators [5]. The overexpression of miRNAs and their target genes have allowed assignment of developmental roles in embryonic, vegetative, and floral organ boundary formation [33, 34], such as miR159, miR164, miR165/166, miR172, and miR319 families. It has demonstrated that miR164 directly regulates NAC domain genes for making the function of normal plant morphogenesis and normal embryonic [34]. In the present study, there was no miR164 detected, but miR164c was expressed in three samples and higher expression level in abnormal embryo (Table S2). Previous study showed the highest expression of miR164c in normal embryo at 18 DAP when the male was diploid *C. nankingense* [24], suggesting that miR164c may have the important regulatory role at the late stage of embryonic development, and the lower expression level of miR164c in normal embryo at 18 DAP may be beneficial to chrysanthemum embryo development normally. Similar situation happened in miR159a; there was no miR159 detected in chrysanthemum embryo, but the miR159a was differentially expressed in normal and abnormal embryo at 18 DAP, suggesting the connected function with miR164a during chrysanthemum embryonic development.

miR172 showed the regulatory function for early flowering and floral organ identity defects by regulating *AP2* and *TOE* [35]. miR172c was detected in this study, and the expression was zero in samples of NE12 and AE18, which means that miR172c mainly mediates gene expression in postembryonic development and these genes may promote embryo maturation. *DCL1* was a key gene for embryo development by embryo lethality, which was regulated by miR163 [36, 37]. However, there was no miR163 identified in chrysanthemum embryo whether the cross has paternal chromosome doubling or not. This result exhibits that *DCL1* was not regulated by miR163 and participated in embryonic lethality in chrysanthemum.

### 4.3. miRNA-Mediated Target Genes in Chrysanthemum Embryo

Research in the last decade has demonstrated that miRNAs make the crucial roles during plant embryogenesis by regulating various genes and pathways [38]. It contains the process of spatial control of differentiation, regulation of auxin responses, and temporal control of differentiation [5]. A study on *miR160*-resistant *ARF17* transgenes showed the defected cotyledons, suggesting that miR160 negatively regulated genes involved in auxin signaling that is critical for proper development of the embryo and cotyledons [39]. Auxin response is a critical biological pathway for embryonic development, and it has also been reported in chrysanthemum [22]. In the present study, miR160 was identified in NE12 and NE18, highly expressed in NE18 (Table S2). The result of target gene prediction showed that the targets of miR160 were auxin response factor with differential expression among samples (Table 2). In the late embryonic development (18 DAP), the target genes (unigene9514) of auxin response factor downregulated compared with NE12. Besides, there are a portion of target genes involved in energy metabolism according to Nr/Nt annotation, and their expression was downregulated in an abnormal embryo (Table 2). Transcriptome provided the evidence of importance of energy synthesis for normal embryo development [22]; here, these identified target genes were regulated by miRNAs, such as miR414, miR2661, and miR5021, also support the significance of energy metabolism for chrysanthemum embryo development.

## 5. Conclusion

Polyploid breeding will pay more attention from genomic research in the future as rapid advances in the next generation sequencing technology, which makes unprecedented opportunities to explore and understand the regulatory of genomic or transcriptomic changes. As a critical regulatory factor, the function of miRNAs has attracted a lot of attention during plant growth and development. In chrysanthemum distant hybridization, breeders always faced the barriers existed in embryo development. The present study provided some explanation about the embryo abortion and the miRNAs related to embryo development even their target genes. We propose that late embryonic miRNAs, especially miR164a, regulate NAC transcription factor and thereby affect the embryonic development. miR160 mediated the auxin response, and miR414, miR2661, and miR5021 regulate the genes involved in energy metabolism; together, they regulate the embryo development in chrysanthemum hybridization.

## Figures and Tables

**Table 1:** Quantity and type distribution of small RNAs in chrysanthemum embryo.

	NE12				NE18				AE18			
Category	Unique sRNAs	Percent (%)	Total sRNAs	Percent (%)	Unique sRNAs	Percent (%)	Total sRNAs	Percent (%)	Unique sRNAs	Percent (%)	Total sRNAs	Percent (%)
Total	3,864,037	100	11,535,216	100	3,780,667	100	10,805,231	100	3,734,164	100	11,220,674	100
Mapping to genome	371,560	9.62	2,251,582	19.52	379,579	10.04	2,194,212	20.31	384,290	10.29	2,383,150	21.24
miRNA	9900	0.26	410,884	3.56	9192	0.24	343,417	3.18	9871	0.26	360,354	3.21
rRNA	57,663	1.49	547,936	4.75	68,080	1.80	662,520	6.13	81,807	2.19	852,436	7.60
snRNA	1423	0.04	3088	0.03	1475	0.04	3034	0.03	1564	0.04	3278	0.03
snoRNA	421	0.01	757	0.01	461	0.01	789	0.01	613	0.02	1205	0.01
tRNA	7221	0.19	72,063	0.62	7332	0.19	97,766	0.90	9506	0.25	118,373	1.05
Unannotated	3,787,409	98.02	10,500,488	91.03	3,694,127	97.71	9,697,705	89.75	3,630,803	97.23	9,885,028	88.10

**Table 2:** Target genes identified in chrysanthemum embryo.

miRNA	ID of target genes	NE12_FPKM	NE18_FPKM	AE18_FPKM	Nr/Nt annotation
miR159a	Unigene13056_All	22.8776	14.0501	14.2128	50S ribosomal protein
miR160a	CL8743.Contig2_All	5.2658	7.4008	5.3906	Auxin response factor
miR160a	Unigene9514_All	17.7131	8.5644	12.0923	Auxin response factor 10
miR164c	Unigene24220_All	1.0397	6.6612	11.3245	NAC transcription factor
miR166a	Unigene33904_All	2.1379	3.8597	1.4223	Homeobox-leucine zipper protein
miR167c-3p	CL15007.Contig2_All	16.0571	29.3484	35.2898	AMP-activated protein kinase
miR169b	CL3042.Contig2_All	2.9511	8.0941	7.2676	Nuclear transcription factor Y
miR169b	CL3042.Contig3_All	2.5107	7.0631	5.6611	Nuclear transcription factor Y
miR169b	Unigene26767_All	11.8519	30.0804	26.5044	Nuclear transcription factor Y
miR172c	Unigene23005_All	1.827	3.0981	4.1985	Floral homeotic protein APETALA2
miR2653a	Unigene7445_All	0.4387	4.6181	26.8109	*Solanum lycopersicum* RAP2.6-like protein gene
miR2661	Unigene35846_All	1.8822	4.5798	0.9556	ATP binding protein
miR2868	Unigene46_All	10.1108	17.9793	28.7056	Protein NLP6-like
miR396a-3p	CL936.Contig4_All	10.6134	9.8829	7.7245	MAK16 protein-like protein
miR397a	Unigene30076_All	29.7323	13.9125	13.4372	Glucan endo-1,3-beta-glucosidase 7-like
miR397a	Unigene4252_All	1.9732	5.4082	2.1149	*Glycine max* laccase-4-like
miR403	CL516.Contig5_All	26.1176	43.3925	45.8309	Argonaute protein group
miR414	CL4550.Contig3_All	12.7195	19.889	8.8036	ADP-glucose pyrophosphorylase small subunit 2
miR414	CL2586.Contig3_All	2.6852	5.6627	7.2585	ATP binding protein
miR414	CL12204.Contig1_All	35.1412	15.8001	13.8476	CAPIP2
miR414	Unigene19498_All	3.0041	2.9203	1.0519	F-box protein At5g07610
miR414	Unigene12916_All	31.2274	55.6863	66.4644	F-box/LRR-repeat protein
miR414	Unigene10707_All	0.158	2.7039	4.4112	Glutamate receptor 2.3
miR414	CL7369.Contig4_All	15.1331	11.8677	23.1584	Glycine max surfeit locus protein 2-like
miR414	CL4274.Contig1_All	0.1141	5.574	1.6227	Histone H2B.2
miR414	Unigene32715_All	2.8017	9.3813	2.9311	Minichromosome maintenance factor
miR414	CL7491.Contig4_All	56.838	23.8057	28.7017	NA
miR414	CL7491.Contig3_All	40.3425	15.2757	19.8302	NA
miR414	CL7491.Contig1_All	40.1769	18.4149	17.7948	Proline-rich protein
miR414	CL11069.Contig1_All	1.594	2.2067	4.0463	Protein NLP6-like
miR414	Unigene30228_All	1.7963	4.9734	9.0057	Uncharacterized protein
miR414	Unigene31458_All	33.3706	106.7705	151.1735	Uncharacterized protein
miR414	Unigene28799_All	0.6622	1.2	2.6895	Uncharacterized protein
miR414	CL15100.Contig1_All	2.7599	11.7908	10.9824	Uncharacterized protein
miR414	CL5368.Contig4_All	23.3918	71.719	84.0485	Uncharacterized protein
miR414	CL5528.Contig1_All	3.1358	4.9787	1.9595	Uncharacterized protein
miR414	Unigene35998_All	2.9444	0.2695	0.8154	Uncharacterized protein
miR414	Unigene19911_All	3.7645	2.6847	0.7964	Uncharacterized protein
miR414	Unigene25838_All	2.1786	8.7075	24.971	Transcription factor WRKY
miR414	Unigene27592_All	109.2811	89.6706	72.8937	ATP synthase subunit O
miR5021	CL13854.Contig1_All	20.2409	11.1804	9.5598	ATP binding protein
miR5021	Unigene49221_All	0.4791	0.8039	3.8915	NA
miR5021	Unigene8421_All	8.1632	6.9848	3.84	PAPS-reductase-like protein
miR5021 miR5293	CL11776.Contig1_All	39.399	34.2386	34.2098	Uncharacterized protein
miR5049c	CL559.Contig1_All	33.8479	33.5268	45.5856	Chrysanthemum x morifolium DREBa
miR5049c miR5052a	Unigene1703_All	1.4503	2.1902	4.4178	NA
miR5077	CL14985.Contig3_All	2.6774	4.7455	5.6638	Global transcription factor group
miR5227	CL4864.Contig1_All	58.2913	17.2209	11.1415	ATP-citrate synthase beta chain protein
miR5270a	Unigene3852_All	2.5276	5.0261	13.2176	Uncharacterized protein
miR5293	CL7596.Contig2_All	999.1282	2027.7828	2112.3254	Dehydrin family protein
miR5293	Unigene16818_All	9.169	7.1012	3.1716	Glycine max reticulon-like protein B21-like
miR5293	Unigene31007_All	34.275	32.3294	106.0944	Uncharacterized protein
miR5298b	Unigene4147_All	12.7734	20.3587	42.7602	Uncharacterized protein
miR5298b miR5380a	CL5088.Contig2_All	22.2504	20.7529	20.0829	Nucleosome assembly protein 1-like
miR5380a	Unigene7407_All	6.0977	88.9232	149.3867	Cytochrome P450
miR5380a	Unigene19991_All	0.6069	3.055	1.0955	Ferric reduction oxidase
miR5215	CL965.Contig5_All	0	0.5661	1.5225	Disease-resistance protein NRSA1
miR5215 miR5373	Unigene21756_All	5.9645	5.6689	6.2018	ATP binding protein
miR5373	CL561.Contig3_All	39.0762	27.122	25.8912	Glycyl-tRNA synthetase 1
miR5640	Unigene11631_All	1.3529	38.198	4.7781	Glutathione S-transferase 1
miR5641	Unigene3971_All	21.8502	14.3017	11.807	Glycosyl transferase
miR5641	Unigene7799_All	1.2821	1.6384	5.4077	RPL5A-related protein
miR6111-5p	Unigene29644_All	5.3413	11.3752	17.9804	Cytokinin-regulated kinase 1
miR6220-3p miR818a	CL1844.Contig1_All	9.9143	3.9489	3.9549	Benzoyl CoA benzoic acid benzoyltransferase
miR845b-5p	Unigene2353_All	10.1133	40.3692	61.9776	NA
miR858b	Unigene2183_All	19.3548	9.2995	5.5141	Transcription factor MYB11

## Supplementary Materials

Supplementary 1 Table S1: primers used to verify the expression of miRNAs and target genes.

Supplementary 2 Table S2: expression level of miRNAs identified in chrysanthemum embryo.

Supplementary 3 Table S3: all identified target genes in chrysanthemum embryo.

Supplementary 4 Table S4: KEGG annotation of target genes.

Supplementary 5 Table S5: annotation of all target genes.
